# Evaluating understandability and actionability of online education materials for home-care patients with COVID-19 in Japan

**DOI:** 10.1186/s13104-023-06570-1

**Published:** 2023-10-25

**Authors:** Emi Furukawa, Tsuyoshi Okuhara, Hiroko Okada, Naomi Sawada, Takahiro Kiuchi

**Affiliations:** 1 Department of Health Communication, The University of Tokyo Graduate School of Medicine, Tokyo, Japan; 2 Department of Health Communication, School of Public Health, The University of Tokyo, 7-3-1 Hongo, Bunkyo-ku, Tokyo, 113-8655 Japan

**Keywords:** COVID-19, Patient education, Health communication

## Abstract

**Objective:**

In Japan, educational materials on the home care of coronavirus disease 2019 (COVID-19) were developed owing to limited access to medical care during the pandemic. This study quantitatively evaluated the understandability, actionability, natural flow, and readability of 87 materials published by local governments in Japan for patients with COVID-19. Their understandability and actionability were rated using the Japanese version of the Patient Education Material Evaluation Tool for Printed Materials (PEMAT-P). Natural flow and readability were rated using Global Quality Score (GQS) and jReadability, respectively.

**Results:**

Of the 87 materials, 55 (62.1%) were understandable and 33 (37.9%) were actionable according to the PEMAT-P. Regarding understandability, the materials used medical terms without providing definitions and lacked summaries. Regarding actionability, the materials did not demonstrate explicit steps or utilize visual aids to help the readers take action. The mean (SD) of GQS was 3.44 (0.98), indicating a moderate level of naturalness and comprehensiveness of the materials. The mean (SD) score for readability was 2.4 (0.6), indicating a “lower advanced” level. However, challenges regarding the materials’ plain language remained, such as defining medical terms, summarizing the content for understandability, and using charts and tables that encourage patients to act.

**Supplementary Information:**

The online version contains supplementary material available at 10.1186/s13104-023-06570-1.

## Introduction

The coronavirus disease 2019 (COVID-19) outbreak caused a global public health crisis and was declared as a pandemic on March 11, 2020 by the World Health Organization (WHO). By April 1, 2023, approximately 26.5% of the population in Japan (33,462,859 people) had been diagnosed with COVID-19 [1]. According to the WHO, self-isolation and home care was suggested for asymptomatic or mild/moderate COVID-19 patients without risk factors [2]. However, considering the difficulty in accessing medical facilities and communicating directly with healthcare professionals, patients were at an increased risk of being exposed to misinformation. Therefore, information with understandable content on home care and recommendations of suitable actions was urgently required.

Several studies have evaluated the quality of websites associated with COVID-19. Kruse et al. [3] evaluated 141 COVID-19-related materials published by the United States’ academic hospitals and reported that their understandability was good but actionability was poor. Higashi et al. evaluated 50 websites on COVID-19 for patients with cancer in the United States and reported an overall understandability and actionability score of 82%. However, there were persistent difficulties, such as the use of medical terminology and absence of visual aids, which increases the ease of understanding the content [4]. These studies assessed materials explaining general information about COVID-19 from the initial days of the pandemic. Considering that three years have passed since the declaration of the pandemic, the lay public requires tailor-made information. Therefore, messages specific to COVID-19 educational materials include information on ways to manage symptoms without direct access to medical care, measures required in case the disease worsens at home, and ways to prevent the spread of infection in households. In Japan, these messeges distributed from local government were based on guidelines by Ministry of Health, Labor and Welfare. Additionally, many online educational materials were developed to assist patients who were unable to visit medical institutions during quarantine or self-isolation [5]. However, to the best of our knowledge, there was no comprehensive analysis of the quality of Japanese-language material on COVID-19. Hence, our study evaluated whether educational materials on COVID-19 home care in Japan were easily understandable and supported the health behavior of infected individuals.

## Materials and methods

### Study design

#### After revision

The COVID-19-related websites from the local governments (prefectures, ordinance-disignated cities and Tokyo metropolitan districts) were systematically and quantitatively analyzed using web accessibility evaluation tools. The Research Ethics Committee of the University of Tokyo Graduate School of Medicine and Faculty of Medicine waived informed consent for this study because educational materials on the website are publicly accessible and do not involve patient records.

### Study sample

We evaluated educational materials on home care of COVID-19 patients available on local government websites on June 25, 2022. In Japan, COVID-19 patients registered themselves using the Ministry of Health, Labor and Welfare’s Health Center Real-time Information-Sharing System on COVID-19 (HER-SYS) via the website of the local government of their area. Local public health centers monitored patients’ conditions based on the information recorded in the HER-SYS [6]. In the present study, we searched for materials from COVID-19-related portal sites for all prefectures (n = 47), ordinance-designated cities (n = 20), and Tokyo Metropolitan Districts with populations of 500,000 or more (n = 8) to ensure comprehensiveness. The analyzed materials included webpages containing instructions for the home care of COVID-19 patients and leaflets in PDF format that were included on the webpage. All these materials were free to view or download.

### Evaluation methods

#### Understandability and actionability

The Japanese version of the Patient Education Material Evaluation Tool for Printable Materials (PEMAT-P) was used to evaluate the materials’ understandability and actionability [7]. The PEMAT-P evaluates printable materials, i.e., brochures, booklets, and online materials in HTML or PDF format [8]. It is intended to be used by those who provide educational materials to patients or consumers, including healthcare providers, health librarians, and staff of governmental agencies. The PEMAT-P has two subdomains: (1) understandability, which measures how well the written material is understood by health consumers from diverse backgrounds, and (2) actionability, which measures how well consumers can identify what they need to do based on the information provided. It includes 23 items (16 items for understandability and seven for actionability) with a binary scale (agree = 1 or disagree = 0) (Additional file 1). The PEMAT-P understandability and actionability scores are calculated by adding all the points, dividing by the total possible points (excluding not applicable items), and multiplying by 100 to obtain a percentage. The threshold for both subdomains was set at 70% [9].

#### Natural flow and comprehensiveness

Each webpage was rated using the Global Quality Score (GQS) to evaluate the overall natural flow and comprehensiveness of the information on a five-point Likert scale (Additional file 2) [10]. It has been commonly used to evaluate health and medical information websites [11–13]. The GQS consists of a single item, while a score of one point indicates the poorest quality, a score of five points indicates excellent quality.

#### Readability

We additionally used readability, an objective measure to evaluate textual information. The plain text from each webpage was extracted, and any formatting elements that might interfere with readability assessment (headings, symbols, author information, and references) were removed. The text was then assessed using jReadability, an online readability evaluation system [14]. This tool automatically calculates readability based on the average length of sentences, difficulty level of words, proportion of grammatical parts of speech, and types of characters per sentence (Additional file 3).

### Statistical analysis

Descriptive statistics were used to summarize the characteristics of the retrieved websites and calculate PEMAT-P, GQS, and readability scores. Since the PEMAT-P and GQS are subjective measures, we measured inter-rater reliability. Two physicians (EF and NS) with experience in creating patient education materials independently evaluated the material for a quarter of the entire webpages. These webpages were selected by a random number table created in Microsoft Excel. After the inter-rater reliability (Gwet’s AC1) was calculated, EF evaluated the rest of the materials. All statistical analyses were conducted using R (version 4.1.1; 2021-08-10).

## Results

This study included 87 materials from 60 local governments. Table 1 shows the demographics of local governments. Overall, 55 (62.1%) materials were understandable and 33 (37.9%) were actionable according to the PEMAT-P. The mean understandability and actionability scores were 73.2% and 62.6%, respectively. PEMAT-P scores between groups were shown in Additional file 4.

**Table 1:** Characteristics of the included local governments

	n	%
Category of local government		
Prefecture	43	71.7
Ordinance-designated cities	14	23.3
Cities in Tokyo Metropolitan Districts	3	5.0
Population		
less than 1,000,000	19	31.7
1,000,000–5,000,000	33	55.0
more than 5,000,000	8	13.3
Culmulative infection rate(per 100,000)		
less than 5,000	17	28.3
5,000–10,000	34	56.7
more than 10,000	9	15.0
2nd COVID-19 vaccination rate		
less than 70%	1	1.7
70%-74.9%	19	31.7
75%-79.9%	32	53.3
more than 80%	8	13.3

Note: Population data was extracted as of October 1, 2021

Data of culmulative infection rate(per 100,000)and 2nd COVID-19 vaccination rate was extracted as of June 25, 2022

Table 2 presents the scores for each PEMAT-P item. The highest percentage of the materials met the criteria for Item 1 on understandability: “The material made its purpose completely evident from the beginning (99%),” while the lowest percentage was for Item 10: “The material provided a summary (16%).” For actionability, most materials met the criteria for Item 19: “The material clearly identified at least one action that the user could perform (100%),” while the least materials met the criteria for Item 24: “The material explained how to use charts, graphs, tables, or diagrams to take actions (36%).”

**Table 2:** The mean score for each of the PEMAT-P item

Item #	Item	Item score	
		mean	SD
UNDERSTANDABILITY		73.2%	15.4%
TOPIC: CONTENT			
1	The material makes its purpose completely evident from the beginning	0.99	0.11
2	The material does not include information or content that distracts from its purpose	0.66	0.48
TOPIC: WORD CHOICE & STYLE			
3	The material uses common, everyday language	0.6	0.49
4	Medical terms are defined when they are used	0.46	0.5
TOPIC: USE OF NUMBERS			
5	Numbers appearing in the material are clear and easy to understand	0.99	0.11
6	The material does not expect the user to perform calculations	0.82	0.39
TOPIC: ORGANIZATION			
7	The material breaks or “chunks” information into short sections	0.83	0.38
8	The material’s sections have informative headers	0.94	0.24
9	The material presents information in a logical sequence	0.69	0.47
10	The material provides a summary	0.16	0.37
TOPIC: LAYOUT & DESIGN			
11	The material uses visual cues (e.g., arrows, boxes, bullets, bold, larger font, highlighting) to draw attention to key points	0.82	0.39
TOPIC: USE OF VISUAL AIDS			
14	The material uses visual aids whenever they could make content more easily understood (e.g., illustration of healthy portion size)	0.76	0.43
15	The material’s visual aids reinforce rather than distract from the content	0.96	0.2
16	The material’s visual aids have clear titles or captions	0.63	0.49
17	The material uses illustrations and photographs that are clear and uncluttered	0.71	0.46
18	The material uses simple tables with short and clear row and column headings	0.92	0.28
ACTIONABILITY		62.6%	25.4%
19	The material clearly identifies at least one action the user can take	1	0.00
20	The material addresses the user directly when describing actions	0.98	0.15
21	The material breaks down any action into explicit steps	0.53	0.5
22	The material provides a tangible tool (e.g., menu planners, checklists) whenever it could help the user take action	0.45	0.5
23	The material provides simple instructions or examples of how to perform calculations	0.67	0.49
24	The material explains how to use the charts, graphs, tables, or diagrams to take actions	0.36	0.48
25	The material uses visual aids whenever they could make it easier to act on the instructions	0.43	0.5

PEMAT items were scored with a binary scale (agree = 1 or disagree = 0). The PEMAT-P understandability and actionability scores were calculated by adding all the points, dividing by the total possible points (excluding not applicable items), and multiplying by 100 to obtain a percentage

The mean (SD) score of GQS was 3.44 (0.98), and range was 1 to 5. The mean score indicates a moderate level of naturalness and comprehensiveness of the materials. High inter-rater reliability was obtained for each of the PEMAT-P items (Gwet’s AC1 = 0.71-1.00) and GQS (Gwet’s AC1 = 0.76). The mean (SD) score for readability was 2.4 (0.6), indicating a “lower advanced” level. This score indicates that readers should have language skills to understand the complex structures found in the Japanese literature to comprehend the material.

## Discussion

More than half of the included materials met the criteria for understandability; however, less than 40% of the materials were rated as actionable. The PEMAT-P scores in this study did not deviate much from previous studies [3, 4, 15–17].

This study confirmed the issues identified in previous studies which analyzed COVID-19 home care materials developed by governmental agencies [3, 4, 15–18]. First, concerning understandability, the materials used medical terms, such as “SpO_2_,” “specimen collection,” “risk factors,” “underlying disease,” and “mild/severe disease,” without including definitions or explanations. Additionally, most materials did not include summaries, making it difficult for readers to understand the main points at a glance [4]. Second, concerning actionability, the materials did not demonstrate explicit steps to help readers take action [4, 16]. Moreover, they did not use visual aids (i.e., tables, charts, illustrations, or diagrams) in situations that could promote the recommended actions [18]. Moreover, they did not use visual aids (i.e., tables, charts, illustrations, or diagrams) in situations that could promote the recommended actions [18]. This reflects a lack model [19], which states, “if the experts fill in the gaps in citizens’ knowledge, they will accept what the experts said”. However, we cannot expect the audience to act when they are only provided with knowledge. It is necessary to deliver messages about the behaviors in a way that increases self-efficacy for the audience, as in the actionability items listed in PEMAT.

The comprehensiveness and natural flow of the materials were moderate, and there were no substantial quality gaps between local governments. The readability level of the materials was lower at the advanced level. These findings were consistent with those of previous studies examining online information on COVID-19, which showed that the analyzed webpages mostly required a higher reading level than the recommended six-grade reading level [20–24]. This poor readability level is concerning because laypeople considered materials from local governments as a major source of health information during the COVID-19 pandemic.

In conclusion, the COVID-19 home care materials developed by the Japanese local government satisfied the criteria of understandability and actionability. The comprehensiveness and natural flow of the materials were moderate. However, as in previous studies, challenges were identified in defining medical terms, summarizing the content for understandability, and the use of charts and tables that could encourage patients to take action. The materials were somewhat difficult for non-native Japanese speakers or those without higher education to comprehend. While access to medical care was limited during the COVID-19 pandemic, COVID-19 home care information should be disseminated to the general public in an understandable and actionable manner.

### Limitations

While patients with COVID-19 may use resources outside public institutions, this study only evaluated materials from local governments. Additionally, the understandability and actionability of educational materials were measured at one distinct timepoint in this study. Therefore, it does not reflect the most recent data. Hence, further studies are required to evaluate the latest data and conduct a longitudinal comparative study to determine improvements. The Japanese version of PEMAT has been verified for predictive validity through surveys of the general public. However, the items of the PEMAT do not fully reflect the patient’s perspective. Therefore, the opinions of the audience should be evaluated elsewhere. Future research is needed to qualitatively assess how audience feel about the materials, or how they likely to take actions recommended in the materials.

### Electronic supplementary material

Below is the link to the electronic supplementary material.

Supplementary Material 1

Supplementary Material 2

Supplementary Material 3

Supplementary Material 4

## Data Availability

The datasets used and/or analyzed during the current study are available from the corresponding author on reasonable request.

## Abbreviations

WHO: The World Health Organization
COVID-19: The coronavirus disease 2019
HER-SYS: The Ministry of Health, Labor and Welfare’s Health Center Real-time Information-Sharing System on COVID-19
PEMAT-P: Patient Education Material Evaluation Tool for Printed Materials
GQS: The Global Quality Score
